# Parameters matter: modulating cytokines using nerve stimulation

**DOI:** 10.1186/s42234-020-00049-1

**Published:** 2020-06-26

**Authors:** Bruno Bonaz

**Affiliations:** University Grenoble Alpes, Inserm, U1216, Grenoble Institute Neurosciences and Division of Hepato-Gastroenterology, CHU Grenoble Alpes, 38000 Grenoble, France

## Abstract

The vagus nerve-based inflammatory reflex regulates inflammation and cytokine release. Recent successful clinical trials using implantable bioelectronic devices to modulate the inflammatory reflex in patients with rheumatoid arthritis and inflammatory bowel disease have demonstrated the efficacy of targeting neural circuits as an efficient alternative to drug treatments. However, the optimal vagus nerve stimulation parameters to achieve efficacious symptomatic relief for inflammation are still unknown. In this issue of Bioelectronic Medicine, Tsaava et al. tested whether altering these electrical stimulation parameters would change circulating cytokine levels in healthy mice. They found that specific combinations of parameters produced significant increases in serum TNF while other parameters selectively lowered serum TNF levels, as compared to sham stimulated mice. These results have considerable implications for determining the optimal stimulation parameters to better treat common conditions and diseases that involve immune regulation.

## What you need to know

### Background and context

Recent successful clinical trials using implantable bioelectronic devices that modulate the inflammatory reflex have reported efficacy of vagus nerve stimulation in the treatment of inflammatory diseases. However, the optimal neurostimulation parameters to achieve significant cytokine changes are still unknown.

### New findings

In healthy mice without inflammation, specific combinations of pulse width, pulse amplitude, and frequency produce significant increases of the pro-inflammatory cytokine TNF but other parameters selectively lower serum TNF levels. Serum levels of the anti-inflammatory cytokine IL-10 were also significantly increased by selective parameters but remained unchanged with others.

### Impact

These findings provide guidance for design of future studies on the neural regulation of inflammation based on the selection of optimal parameters to relieve conditions and improve organ function.

Bioelectronic medicine is based on neuromodulation of the nervous system restoring organ functions and health with less adverse effects than drugs, thus minimizing adherence issues (Olofsson and Tracey 2017). Pilot clinical studies using implantable bioelectronic devices targeting a vagus nerve reflex circuit, the inflammatory reflex, have reported efficacy in the treatment of patients with rheumatoid arthritis and inflammatory bowel disease (Koopman et al. 2016; Bonaz et al. 2016). Accordingly, understanding the neuromodulation parameters for stimulation of the vagus nerve (VN), the longest nerve of the organism involved in the control of visceral functions (Grundy 1988), is important (Bonaz et al. 2017).

VN stimulation (VNS) is approved for the treatment of drug-resistant epilepsy and well tolerated (Boon et al. 2018). Two types of VNS are presently performed in humans (Yuan and Silberstein 2016) either *i)* invasive with surgical implantation of an electrode around the left VN linked to a neurostimulator positioned under the collarbone or *ii)* non-invasive through transcutaneous cervical (on the path of the VN) or auricular stimulation, based on the innervation of the cymba conchae by the auricular branch of the VN (Peuker and Filler 2002). The classical parameters of VNS, output current, pulse width, pulse frequency, and duty cycle (i.e. ON/OFF time) determine the total amount of electrical energy delivered to the VN during treatment (Groves and Brown 2005). The current guidelines of invasive VNS in epilepsy are: output currents between 0.25 and 3.5 mA, pulse frequencies between 20 and 30 Hz, pulse width between 250 μs to 500 μs, standard duty cycle 30 s ON/5 min OFF (Groves and Brown 2005).

However, the optimal parameters of VNS to achieve efficacious inflammation-related symptomatic relief by recruiting the appropriate fibers within the VN are still unknown. In this context, Tsaava et al. (2020), in a paper published in the present issue of Bioelectronic Medicine, tested whether altering the parameters of invasive VNS of the left cervical VN would change circulating cytokine levels of healthy mice in the absence of increased inflammation.

While VNS is able to decrease serum TNF in inflammatory conditions, its effect in normal conditions was previously unknown. Indeed, the VN mediates anti-inflammatory actions through its afferents, activating the hypothalamic-pituitary adrenal axis to release anti-inflammatory corticosteroids by the adrenal glands. Vagal efferents also have an anti-inflammatory role through the cholinergic anti-inflammatory pathway (CAP) (Borovikova et al. 2000). The VN interacts with the sympathetic splenic nerve via the celiac ganglion to inhibit the release of TNF by splenic macrophages. This effect is mediated through the link of norepinephrine on β2-adrenergic receptors of splenic lymphocytes that release ACh that suppresses TNF release by macrophages through interaction with alpha7-nicotinic ACh receptors expressed on macrophages (Wang et al. 2003; Rosas-Ballina et al. 2008). Together these studies provide a rationale for targeting neural circuits to regulate immune responses with potential therapeutic implications in the domain of TNF-mediated diseases, such as rheumatoid arthritis and inflammatory bowel disease (Crohn’s disease and ulcerative colitis) as demonstrated successfully in pilot studies using VNS (Bonaz et al. 2016; Koopman et al. 2016). The sympathetic pathway, through the splanchnic nerves interacting with the spleen, has also shown anti-inflammatory effects and could be a target of bioelectronic medicine (Brinkman et al. 2019).

Tsaava et al. (2020) tested a set of electrical stimulation parameters and measured serum cytokine levels in healthy mice with the following stimulation parameters: 4 min duration, pulse width (50 μs, 250 μs), amplitude (50 μA, 200 μA, and 750 μA), and frequency (30 Hz, 100 Hz). Sham operated mice underwent a similar surgery to expose the VN, but did not receive any electrical stimulation. Two hours after stimulation, blood samples were collected by cardiac puncture following euthanasia and serum was analyzed on multiplex cytokine immunoassay plates to quantify levels of IFN-γ, IL-1β, IL-2, IL-4, IL-5, IL-6, CXCL1, IL-10, IL-12p70, and TNF. They found that specific combinations of pulse width, pulse amplitude, and frequency produced significant increases of the pro-inflammatory cytokine TNF, while other parameters selectively lowered serum TNF levels, as compared to sham stimulated mice. Indeed, stimulation at the short pulse width 50 μs at 30 or 100 Hz pulse and 200 or 750 μA amplitude produced a significant decrease in TNF. When increasing the pulse width to 250 μs they observed a significant increase in serum TNF levels at 30 Hz and 750 μA, compared to sham mice, while stimulation with a pulse width of but not at 250 μs and 100 Hz. These data suggest that specific stimulation parameters can alter serum TNF in a bidirectional manner. In addition, serum IL-10 levels were significantly increased at 50 μs pulse width and 30 Hz at both the 50 μA and 750 μA amplitudes but remained unchanged at 50 μs pulse width and 100 Hz stimulation. A significant increase in IL-10 was also observed with the longer 250 μs pulse width at 750 μA amplitude for both the 30 Hz and 100 Hz frequencies and at 100 Hz with a 50 μA amplitude. Thus, specific parameters have a different effect on serum IL-10, compared to TNF. They also showed that VNS exert differential effects on the modulation of other cytokines such as serum levels of IL-6, which were increased across a wide range of stimulation parameters for both the short 50 μs and long 250 μs pulse widths. The authors conclude that VNS parameter selection is critically important for the modulation of cytokines via the cervical VN and that specific cytokines can be increased by electrical stimulation in the absence of inflammation.

The authors also showed that alteration of stimulation parameters resulted in differential effects on heart rate, with longer pulse width increasing the likelihood of recruiting cardiac innervating B-fibers in the mouse VN. Indeed, the right VN innervates the sinoatrial node, involved in the pacemaker function of the heart, whereas the left VN innervates the atrioventricular node, regulating the force of contraction of the heart muscle with less influence over heart rate. Prior work has shown that VNS of the right, compared to the left VN, caused a greater reduction in heart rate whereas stimulation of the left VN had no effect on heart rate (Woodbury and Woodbury 1990) so that, in experimental and clinical conditions VNS is classically performed on the left VN.

Importantly, these results argue that serum cytokines may be controlled by specific fiber sets and firing patterns. The specific VN fibers mediating the inflammatory reflex are not known. The VN contains A-, B-, and C-fibers, defined in accordance to their conduction velocity, which, in myelinated fibers, is proportional to their size (Erlanger and Gasser 1930). The most numerous fibers are the afferent C-fibers (65–80% in the cat) (Woodbury and Woodbury 1990). The types of fibers play different physiological roles: *i)* vagal A-fibers are the largest and myelinated fibers and carry afferent visceral information and motor input, *ii)* vagal B-fibers are small and myelinated fibers carrying parasympathetic input, and *iii)* vagal C-fibers are small and unmyelinated and carry afferent visceral information. Antiepileptic property of VNS was supposed to be related to vagal C-fibers, but their destruction did not alter VNS-induced seizure suppression in rats (Krahl et al. 2001) thus suggesting that seizure suppression results from activation of vagal A- and B-fibers.

Patients under VNS for epilepsy may experience improved seizure reduction by increasing the frequency and/or duty cycle of stimulation while VNS for heart failure is limited by the inability to activate the nerve fibers mediating therapeutic benefit without co-activation of side effect-inducing fibers (Musselman et al. 2019). However, vagal anti-inflammatory signaling is specific and dissociable from heart rate regulation (Huston et al. 2007). The dissociability and lower activation threshold of vagal anti-inflammatory signaling indicate that A fibers, which have the lowest activation threshold, do not seem to participate in heart rate regulation but may fulfill the role of CAP fibers.

The results of Tsaava et al. (2020) indicate that the refinement of VNS parameters is important in the neuromodulation of immunological responses mediated through vagal signaling. This is the first demonstration that specific stimulation parameters can be used to increase serum TNF and IL-10 levels. However, this study was performed in healthy animals and it will be interesting to assess whether these data can be extrapolated to disease conditions. Other experimental studies are warranted in inflammatory conditions before translating them to humans, with different inflammatory pathologies in particular. In the near future, the development of bioelectronic devices should be integrated with optimal stimulation parameters to better treat specific conditions and diseases.

## Data Availability

Not applicable.

## Abbreviations

Ach: Acetylcholine
CAP: Cholinergic anti-inflammatory pathway
CXCL1: Chemokine (C-X-C motif) ligand 1
IFN: Interferon
IL: Interleukin
TNF: Tumor necrosis factor
VN: Vagus nerve
VNS: Vagus nerve stimulation
